# Structural and functional features of phytoene synthase isoforms PSY1 and PSY2 in pepper Capsicum annuum L. cultivars

**DOI:** 10.18699/VJ20.663

**Published:** 2020-11

**Authors:** E.A. Dyachenko, M.A. Filyushin, G.I. Efremov, E.A. Dzhos, A.V. Shchennikova, E.Z. Kochieva

**Affiliations:** Federal Research Centre “Fundamentals of Biotechnology” of the Russian Academy of Sciences, Moscow, Russia; Federal Research Centre “Fundamentals of Biotechnology” of the Russian Academy of Sciences, Moscow, Russia; Federal Research Centre “Fundamentals of Biotechnology” of the Russian Academy of Sciences, Moscow, Russia; Federal Research Centre “Fundamentals of Biotechnology” of the Russian Academy of Sciences, Moscow, Russia Federal Scientific Vegetable Center, VNIISSOK, Moscow region, Russia; Federal Research Centre “Fundamentals of Biotechnology” of the Russian Academy of Sciences, Moscow, Russia; Federal Research Centre “Fundamentals of Biotechnology” of the Russian Academy of Sciences, Moscow, Russia

**Keywords:** carotenogenesis, Capsicum annuum, pepper fruits, fruit ripening, fruit pigmentation, каротиногенез, Capsicum annuum, плоды перца, созревание плодов, окраска плода

## Abstract

The fruits of various pepper cultivars are characterized by a different color, which is determined by the pigment ratio; carotenoids dominate in ripe fruits, while chlorophylls, in immature fruits. A key regulator of carotenoid biosynthesis is the phytoene synthase encoded by the PSY gene. The Capsicum annuum genome contains two isoforms of this enzyme, localized in leaf (PSY2) and fruit (PSY1) plastids. In this work, the complete PSY1 and PSY2 genes were identified in nine C. annuum cultivars, which differ in ripe fruit color. PSY1 and PSY2 sequence variability was 2.43 % (69 SNPs) and 1.21 % (36 SNPs). The most variable were PSY1 proteins of the cultivars ‘Maria’ (red-fruited) and ‘Sladkij shokolad’ (red-brown-fruited). All identified PSY1 and PSY2 homologs contained the phytoene synthase domain HH-IPPS and the transit peptide. In the PSY1 and PSY2 HH-IPPS domains, functionally significant sites were determined. For all accessions studied, the active sites (YAKTF and RAYV), aspartate-rich substrate-Mg^2+^-binding sites (DELVD and DVGED), and other functional residues were shown to be conserved. Transit peptides were more variable, and their similarity in the PSY1 and PSY2 proteins did not exceed 78.68 %. According to the biochemical data obtained, the largest amounts of chlorophylls and carotenoids across the cultivars studied were detected in immature and ripe fruits of the cv. ‘Sladkij shokolad’ and ‘Shokoladnyj’. Also, ripe fruits of the cv. ‘Nesozrevayuschij’ (green-fruited) were marked by significant chlorophyll content, but a minimum of carotenoids. The PSY1 and PSY2 expression patterns were determined in the fruit pericarp at three ripening stages in ‘Zheltyj buket’, ‘Sladkij shokolad’, ‘Karmin’ and ‘Nesozrevayuschij’, which have different ripe fruit colors: yellow, red-brown, dark red and green, respectively. In the leaves of the cultivars studied, PSY1 expression levels varied significantly. All cultivars were characterized by increased PSY1 transcription as the fruit ripened; the maximum transcription level was found in the ripe fruit of ‘Sladkij shokolad’, and the lowest, in ‘Nesozrevayuschij’. PSY2 transcripts were detected not only in the leaves and immature fruits, but also in ripe fruits. Assessment of a possible correlation of PSY1 and PSY2 transcription with carotenoid and chlorophyll content revealed a direct relationship between PSY1 expression level and carotenoid pigmentation during fruit ripening. It has been suggested that the absence of a typical pericarp pigmentation pattern in ‘Nesozrevayuschij’ may be associated with impaired chromoplast formation.

## Abstract (ru)

Плоды сортов перца характеризуются различной окраской, которая определяется соотноше- нием пигментов, при этом в спелых плодах доминируют каротиноиды, тогда как в неспелых – хлорофиллы (иногда вместе с антоцианами). Ключевым регулятором биосинтеза каротиноидов является фитоинсинта- за, кодируемая геном PSY. Геном перца Capsicum annuum содержит два гена, кодирующих фитоинсинта- зы, одна из которых локализуется преимущественно в пластидах листа (PSY2), другая – в пластидах пло- да (PSY1). В данной работе были идентифицированы полногеномные последовательности PSY1 и PSY2 у девяти сортов C. annuum, различающихся окраской спелого плода. Вариабельность последовательностей составила 2.43 % (69 SNP) и 1.21 % (36 SNP). Наиболее вариабельны белки PSY1 сортов Мария (красный плод) и Сладкий шоколад (красно-коричневый плод). В последовательностях PSY1 и PSY2 определены фитоинсинтазный домен HH-IPPS и транзитный пептид. Идентифицированы функционально значимые участки в домене HH-IPPS PSY1 и PSY2 анализируемой выборки сортов перца. Для исследуемых образцов области, ограничивающие активные сайты (YAKTF и RAYV), аспартат-богатые субстрат-Mg^2+^-связывающие сайты (DELVD и DVGED) и другие функциональные сайты, консервативны. Транзитные пептиды были более вариабельны, их сходство у белков PSY1 и PSY2 не превышало 78.68 %. Биохимический анализ показал, что наибольшие количества хлорофиллов и каротиноидов среди исследуемой выборки содержатся в не- спелых и спелых плодах сортов Сладкий шоколад и Шоколадный. Значительным содержанием хлорофил- лов, но минимальным – каротиноидов отличались спелые плоды сорта Несозревающий (зеленый плод). Профиль экспрессии генов PSY1 и PSY2 был определен в перикарпе плода на трех стадиях созревания у сортов Желтый букет, Сладкий шоколад, Кармин и Несозревающий, контрастных по окраске спелого пло- да – желтой, коричневой, темно-красной и зеленой соответственно. В листьях исследуемых сортов уровни экспрессии PSY1 значительно варьировали. Для всех сортов был характерен рост транскрипции гена PSY1 по мере созревания плода, при этом в зрелом плоде максимальный уровень транскрипции выявлен у сорта Сладкий шоколад, а самый низкий – у сорта Несозревающий. Транскрипты PSY2 были выявлены не только в листе и незрелом плоде, но и в спелых плодах. Оценка возможной корреляции транскрипции PSY1 и PSY2 с суммарным содержанием каротиноидов и хлорофиллов показала, что имеется прямая зависимость между уровнем экспрессии гена PSY1 и каротиноидной пигментацией плода в процессе созревания. Высказано предположение, что у сорта Несозревающий отсутствие типичного для плодов перца паттерна пигмента- ции перикарпа в процессе созревания может быть связано с нарушениями образования хромопластов.

## Introduction

The genus Capsicum includes, according to various estimates, 30–35 species, five of which are domesticated: C. annuum, C. chinense, C. frutescens, C. pubescens, and C. baccatum (Moscone et al., 2007; Dias et al., 2013). Peppers, both sweet and hot (chili), have a high dietary value as they are rich in antioxidants, including vitamin C, flavonoids, and carotenoids (Sun et al., 2007; Cervantes- Paz et al., 2014).

It is known that primates, including humans, do not synthesize carotenoids de novo, but are in dire need of them, since, for example, β-carotene and α-carotene are precursors of vitamin A. The antioxidant activity of the carotenoids found in carotenogenic fruits and vegetables helps to reduce the risk of various diseases, such as certain types of cancer, age-related eye pathologies and cardiovascular diseases (Howard et al., 2000; Story et al., 2010; Giuliano, 2017). Among vegetable crops, pepper, which fleshy fruits are enriched with various types of carotenoids, is one of the main sources of antioxidants in the human diet. In this regard, the obtaining of new pepper varieties is an important task of modern breeding (Berry et al., 2019; Sun, Li, 2020).

Pepper species have different antioxidant levels, and many breeding programs use the natural variation to identify the characteristics of “exotic” allelic diversity and donors of specific carotenoid spectra. However, at present, closely related accessions of the same species are predominantly used, which often do not have strong phenotypic differences, usually observed when using wild relatives (Berry et al., 2019).

Carotenoids together with chlorophylls and anthocyanins determine the color of pepper fruits. It should be noted that carotenoids are the dominant pigments in ripe pepper fruits, while chlorophylls (sometimes together with anthocyanins) are in immature, growing fruits. In C. annuum cultivars, fruit color depends on the ratio of pigments, as well as on the stage of ripening: from green, yellow, white or purple in unripe fruits (mature fruit stage, MF), to orange, red, dark red, brown and sometimes almost black – in ripe fruits (ripe fruit stage, RF) (Levy et al., 1995; Márkus et al., 1999; Ha et al., 2007). Usually, sweet peppers are harvested at the technical ripeness stage (blanche fruit, intermediate ripe stage, IR), and hot peppers at biological ripeness (RF). The pepper fruit ripening is accompanied by the transition of tissues containing chloroplasts to tissues containing chromoplasts. In chromoplasts, chlorophylls degrade, but the synthesis of carotenoids continues, which, unlike chlorophylls, are able to accumulate in specialized globular structures (Osorio, 2019). This leads to a decrease in the chlorophyll content, the accumulation of carotenoids and, as a consequence, to a change in the ripening fruit color.

Unlike tomato, in ripe fruits of which the main carotenoids are lycopene and β-carotene, in pepper fruits, carotenogenesis goes further – to the formation of xanthophylls; carotenoid spectrum in ripe pepper fruits is represented by major concentrations of red pigments – capsanthin and capsorubin, as well as by various combinations of minor amounts of orange and yellow pigments β-carotene, β-cryptoxanthin, lutein, zeaxanthin, anthraxanthin and violaxanthin (Giuffrida et al., 2013; Mohd Hassan et al., 2019).

Carotenoid pigments are isoprenoid molecules obtained as a result of successive transformations of the universal precursor, isopentenyl pyrophosphate. Several reactions convert this compound into geranylgeranyl pyrophosphate (GGPP), two molecules of which condense head-totail by phytoene synthase to form phytoene, the precursor of all carotenoids (Fraser et al., 2000).

Thus, phytoene synthase is a key regulator of carotenoid biosynthesis, supplying the main substrate – phytoene (Fraser et al., 2000). This enzyme is encoded by the PSY gene, the expression of which is influenced by intermediate and final products of the pathway (Welsch et al., 2003; Kachanovsky et al., 2012; Enfissi et al., 2017). Several types of phytoene synthases have been identified in plants, and phytoene synthase activity depends on the type of enzyme and its intracellular location (Shumskaya et al., 2012). In Arabidopsis thaliana, only one PSY gene was identified (Zhou et al., 2015), while in tomato Solanum lycopersicum, three, and the protein products of these genes have different localization: PSY1 – in fruit plastids, PSY2 – in leaf plastids, PSY3 – in root plastids (Stauder et al., 2018). In pepper C. annuum, two genes are currently known that encode phytoene synthases, one of them is mainly localized in the leaf plastids (PSY2), the other – in the fruit plastids (PSY1) (Thorup et al., 2000; Kilcrease et al., 2015). Accordingly, in tomato and pepper, PSY2 transcripts are mainly present in photosynthetic green tissues, while PSY1 is found in mature fruits of both crops and in tomato flower petals (Giorio et al., 2008; Kilcrease et al., 2015; Berry et al., 2019; Filyushin et al., 2020). However, both phytoene synthases can be transcribed in all plant organs (Stauder et al., 2018).

This study is focused on identifying the genes of phytoene synthases PSY1 and PSY2 in C. annuum cultivars, assessing their intervarietal variability, both structural and functional, as well as possible correlations between the expression of these genes and fruit pigmentation.

## Materials and methods

**Plant material.** Individual plants of nine C. annuum cultivars were used in the research: eight cultivars of sweet pepper (Nesozrevayuschij, Karmin, Shokoladnyj, Sladkij shokolad, Ratunda, Maria, Gogoshary, and Zheltyj buket) and one hot pepper cultivar (Mechta hozyayki) (Table 1). The plants were grown in a greenhouse at the Federal Scientific Vegetable Center (FSVC, Moscow Region).

**Table 1.**
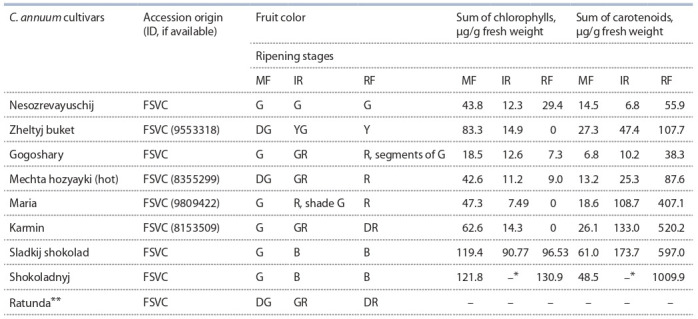
Characteristics of fruits of studied C. annuum cultivars Note. Pepper fruits were analyzed at three stages of ripening: MF – mature green fruit of the final size, IR – intermediate ripe, RF – ripe fruit. Fruit color: G – green, DG – dark green, Y – yellow, YG – yellow green, R – red, DR – dark red, B – brown, GR – green red. Cultivar ID is provided according to the State Register of Breeding Achievements Admitted for Use in RF (https://reestr.gossortrf.ru/). At the time of plant material collection, the IR fruits of cv. Shokoladnyj variety were not found; ** at the time of plant material collection, this cultivar had no fruits at the required ripening stages (in different years, the total carotenoids in Ratunda fruits exceeded that of cv. Shokoladnyj up to two times).

**Identification of the whole genome sequences PSY1 and PSY2.** Genomic DNA was isolated from freshly collected, ground in liquid nitrogen, leaves of each of the analyzed pepper cultivars, according to (Puchooa, 2004). 100 ng of each obtained preparation was used as a template for PSY1 and PSY2 amplification. The amplification primers were previously developed based on the genome sequences of C. annuum PSY1 (LOC107868281 bifunctional 15-cis-phytoene synthase, chromoplastic, Gene ID: 107868281) and PSY2 (LOC107859651 phytoene synthase 2, chloroplastic, Gene ID: 107859651) available in the NCBI database. Amplification of the PSY1 gene was performed with primers CaPSY1F and CaPSY1R (5′-TCAGAATGTCTGTTGCCTTG-3′ and 5′-TCCTG ATTTCATGTTCTTGTAGA-3′), PSY2 – with primers CaPSY2F and CaPSY2R (5′-AGCATGTCTGTTGCTTT GTTG-3′ and 5′-CTTCATTCATGTCTTTGYTAGTG-3′). High precision LongAmp® Hot Start Taq DNA Polymerase (New England Biolabs, Ipswich, MA, USA), C1000 Touch Thermal Cycler (Bio-Rad Laboratories, Inc., Hercules, CA, USA) and the following PCR conditions were used: initial denaturation (94 °C, 10 min); 36 cycles of denaturation (94 °C, 40 s), annealing (56 °C, 40 s) and synthesis (65 °C, 4 min); final completion of the fragments (65 °C, 7 min). Amplified fragments of the expected size were purified from agarose gel using a QIAEX® II Gel Extraction kit (QIAGEN, Hilden, Germany), cloned into the pGEM®-T Easy vector (Promega, Madison, WI, USA), and sequenced (2–4 clones for each sample) on ABI Prism 3730 DNA Analyzer (Applied Biosystems, Waltham, MA, USA).

**Comparative structural analysis of the PSY1 and PSY2.** Alignment and analysis of the obtained nucleotide and amino acid sequences were performed using the MEGA 7.0 (https://www.megasoftware.net/). Known sequences of C. annuum PSY1 (Gene ID: 107868281) and PSY2 (Gene ID: 107859651) were used for comparative analysis. Conserved domains in encoded proteins were determined using the NCBI-CDD (http://www.ncbi.nlm.nih.gov/Structure/cdd/wrpsb.cgi) and UniProtKB (https://www.uniprot.org/). The functional significance of each amino acid (aa) residue substitution was predicted using the PROVEAN (http://provean.jcvi.org/index.php). By radical substitutions is meant those substitutions that can presumably affect the folding of the protein or its functionality.

For cluster phylogenetic analysis of the PSY1 and PSY2 genes identified in pepper cultivars, we also used the PSY gene sequences in C. annuum cv. Zunla 1 (PSY1, NC_ 029980.1:c205334820-205328571; PSY2, NC_029978.1: 142877052-142881261) and C. annuum cv. Valencia (PSY1, GU085273.1). The analysis was performed using the neighbor-joining (NJ) method in the MEGA 7.0 program.

**PSY1 and PSY2 expression pattern in fruits of the analyzed pepper cultivars during ripening.** Total RNA was isolated (RNeasy Plant Mini Kit, QIAGEN, Germany) from fruit pericarp at three developmental stages (MF, IR, and RF). The resulting preparations were purified from DNA impurities (RNase free DNasy set, QIAGEN, Germany), evaluated qualitatively and quantitatively (spectrophotometrically and by electrophoresis in 1.5 % agarose gel), and used for the cDNA synthesis (GoScript™ Reverse Transcription System, Promega, USA).

To determine the PSY1 and PSY2 expression pattern, quantitative real-time PCR (qRT-PCR) was used, which was carried out in three technical replicates with the kit “Reaction mixture for RT-PCR in the presence of SYBR GreenI and ROX” (Syntol LLC, Russia) on a CFX96 Real- Time PCR Detection System (Bio-Rad Laboratories, USA). Primers for qRT-PCR were developed earlier, based on the C. annuum PSY1 (X68017) and PSY2 (XM_016704726.1) mRNA sequences: for PSY1 – PSY1-F and PSY1-R (5′-GT GAAGAGACAGCTGAGATCG-3′ and 5′-TCTCCGG AGTCATTAGCATCG-3′), and for PSY2 – PSY2-F and PSY2-R (5′-AAGGAGTCGCAGAACTGAGC-3′ and 5′-GTCGTTCGCTTCAATCTCATCTAA-3′) (Filyushin et al., 2020). To normalize gene transcription level, the reference gene Actin7 expression and the primers Actin7- F and Actin7-R (5′-CATTGTGCTCAGTGGTGGTTC-3′ and 5′-TCTGCTGGAAGGTGCTAAGTG-3′) were used (Bemer et al., 2012). The qRT-PCR conditions were: 95 °C – 5 min; 40 cycles (95 °C – 15 s, 62 °C – 50 s). The obtained data were statistically processed using the GraphPad Prism v. 7.02 (https://www.graphpad.com).

**The sum of chlorophylls and the sum of carotenoids in fruit pericarp** (together the skin and pulp) were determined spectrophotometrically in chloroform-methanol extracts; the pigment content was calculated using the formulas (Lichtenthaler et al., 1987; Solovchenko et al., 2001), in two biological and three technical replicates.

## Results and discussion

**Characteristics of the PSY1 and PSY2 gene sequences and proteins encoded by them**

Previously, it has been shown that the PSY1 and PSY2 variability may determine the color of the pepper fruit (Cao et al., 2019; Filyushin et al., 2020). Therefore, for this study, nine C. annuum cultivars were selected, which differ in fruit color during ripening: Nesozrevayuschij, Zheltyj buket, Shkoladnyj, Sladkij shokolad, Karmin, Ratunda, Maria, Gogoshary and Mechta hozyayki (see Table 1). Unripe fruit color of all analyzed cultivars was green or dark green, however, the dynamics of color change as they ripen differed among cultivars. In the cv. Nesozrevayuschij, the fruits remained green until biological ripeness, in the cv. Zheltyj buket they were yellow-green at the IR stage and yellow at the RF stage, in cv. Sladkij shokolad and Shkoladnyj, fruits were red-brown at both stages, while the other four cultivars had green-red fruits at the IR stage and red/dark red fruits at the RF stage (see Table 1).

For each of the nine pepper cultivars, the PSY1 and PSY2 gene sequences were determined, starting from the ATG codon (Table 2). The length of the PSY1 gene was 2844 bp in all analyzed cultivars. For comparison, C. annuum cv. Zunla 1 PSY1 available in the NCBI database (Gene ID: 107868281) has the same size, while S. lycopersicum cv. Heinz 1706 (Gene ID: 543988) is longer (3302 bp). The length of the PSY2 gene in the studied cultivars was 2985 bp, with the exception of PSY2 from cv. Mechta hozyayki (2994 bp, due to the 9-nucleotide insert in the second intron) (see Table 2). The C. annuum cv. Zunla 1 PSY2 (Gene ID: 107859651) is also 2985 bp, whereas S. lycopersicum cv. Heinz 1706 PSY2 (Gene ID: 543964) is 3032 bp. The variability of the PSY1 and PSY2 genomic sequences in pepper accessions was 2.43 % (69 SNPs) and 1.21 % (36 SNPs), while 16 and 15 SNPs were localized in exons, respectively. Compared to S. lycopersicum cv. Heinz 1706 PSY1 and PSY2, PSY1 and PSY2 of the pepper cultivars contained 1072/128 and 818/100 (gene/exons) SNPs.

**Table 2.**
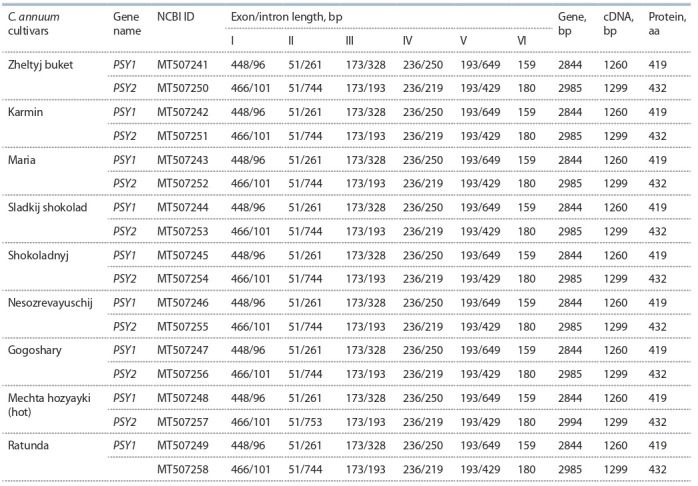
Characteristics of homologous genes PSY1 and PSY2 in C. annuum cultivars

The coding part of the PSY1 and PSY2 genes consisted of six exons and in all studied cultivars was 1260 and 1299 bp, respectively (see Table 2). Found differences in cDNA length were due to the presence of insertions in exons I and VI of PSY2. Most of the identified SNPs were concentrated in exon III of PSY1 (7 SNPs, 43.75 % of all exon substitutions) and in exon VI of PSY2 (6 SNPs, 40.0 %). Exon II of both genes was invariable and the most conserved with respect to the S. lycopersicum cv. Heinz 1706 PSY genes. Exon I of both genes turned out to be the most polymorphic in comparison with the S. lycopersicum PSY genes. Note that since in this work, a limited number of cultivars (nine) were analyzed, the data on gene polymorphism are applicable only to the set of analyzed cultivars.

The PSY1 and PSY2 nucleotide sequences have been translated. The putative proteins PSY1 and PSY2 of all analyzed cultivars were 419 and 432 aa, respectively (see Table 2), contained a conserved phytoene synthase domain HH-IPPS (130–412 and 26–310 aa, according to UniProtKB, and 75–405 and 92–430 aa, according to NCBI- CDD) and the N-terminal transit peptide TP (1–129 and 1–25 aa, according to UniProtKB, and 1–74 and 1–91 aa, according to NCBI-CDD). TP cleavage sites in all possible cases were invariant within the analyzed set of cultivars.

**Fig. 1.**
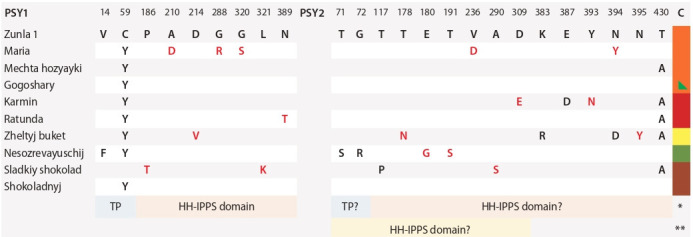
The PSY1 and PSY2 amino acid polymorphism in the studied C. annuum cultivars compared to C. annuum cv. Zunla 1. * According to UniProtKB, ** according to NCBI.

Compared to the C. annuum cv. Zunla 1 and S. lycopersicum cv. Heinz 1706 PSY1 and PSY2, in pepper cultivars, PSY1/PSY2 contained 9/15 and 46/43 aa substitutions, respectively. Out of nine substitutions in PSY1, seven were radical (r) and only two were neutral (n), while all r-substitutions were in the conserved domain, and two n-substitutions were in the transit peptide (Fig. 1). The nC59Y substitution was typical for PSY1 of almost the entire studied set of cultivars, except for the cv. Sladkij shokolad, and all radical substitutions were cultivar-specific. The most variable were PSY1 of cv. Maria and Sladkij shokolad (see Fig. 1).

In the PSY2, of 15 aa substitutions nine were radical. Phytoene synthases PSY2 of cv. Mechta hozyayki, Gogoshary, Ratunda, and Shokoladnyj did not differ from each other or contained nT430A, while each of the other cultivars had one or two r-substitutions in the HH-IPPS domain (see Fig. 1).

The presence of radical aa substitutions in PSY1 and PSY2 of the analyzed cultivars can affect the mature phytoene synthase folding, as well as enzyme ability to interact with protein partners and perform correct catalytic functions. Previously, it was shown that the PSY1 and PSY2 sequences are highly similar (Giorio et al., 2008; Cao et al., 2019). Comparison of the identified C. annuum PSY1 and PSY2 confirmed this observation.

Considering the UniProtKB data on the domain localization, HH-IPPS contains 21 variable sites specific for each of the PSY1 and PSY2 protein groups (see Fig. 1). Also, at the PSY1 domain C-terminus, two deletions, P422–S427del and L429del, were identified. The PSY1 and PSY2 domain sequence was highly conserved (92.86 %).

In contrast to the HH-IPPS domain, the TP sequence was found to be highly variable. In comparison with PSY2 TP, PSY1 TP contained 29 aa substitutions (21.32 % of aligned length). Thus, the identity of TP in PSY1 and PSY2 in the studied pepper cultivars was 78.68 %; also, two insertions (insF31S33 and insG50) and four deletions (PSY2 numbering: N12del, D35del, L57-R62del, and S64- D65del) were identified in the PSY1 sequence. Apparently, differences in TP sequences may be responsible for the specificity of delivery of each of the phytoene synthases to different types of plastids, as was suggested earlier (Cao et al., 2019).

In the PSY1 and PSY2 of the analyzed pepper cultivars, functionally significant sequences were searched. As a result, it was shown that the adjoining regions of active sites (143-YAKTF-147/149-YAKTF-153 and 393-RAYV- 396/399-RAYV-402), aspartate-rich substrate-Mg^2+^- binding sites (173-DELVD-177/179-DELVD-183 and 299- DVGED-303/305-DVGED-309), 18 substrate-binding pockets and 15 catalytic residues are conserved for the cultivars under study. The exceptions were the substitutions rE180G (cv. Nesozrevayuschij, PSY2) at the DELVD site, nD309E (cv. Karmin, PSY2) at the DVGED site, and nG288R (cv. Maria, PSY1, substrate-binding pocket). In all cultivars, the sites F_147_Y_148_/F_153_Y_154_ and A_210_/A_216_ were conserved for both PSY1 and PSY2, with the exception of the rA210D in the cv. Maria PSY1. This composition of functionally important sites determines the similarity of the PSY1 and PSY2 carotenogenic activity level, and, as was shown earlier, the substitution in the A_210_/A_216_ site is not critical, in contrast to F_147_Y_148_/F_153_Y_154_ (Cao et al., 2019). Therefore, found radical substitution rA210D should not significantly affect the cv. Maria PSY1 activity.

**Fig. 2.**
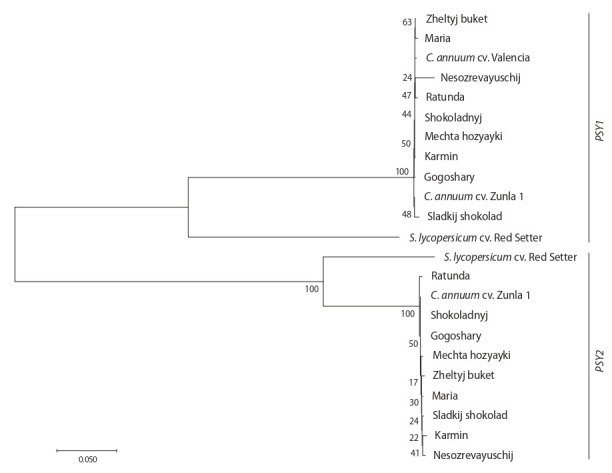
Phylogenetic analysis of the PSY1 and PSY2 full-genome sequences of the studied C. annuum cultivars. The dendrogram was constructed using MEGA 7.0 (NJ method, Tamura–Nei model, bootstrap 1000). For comparison, Solanum lycopersicum cv. Red Setter PSY1 and PSY2 (EF534740.1, EU021055.1) were used.

To confirm the structural similarity of the identified PSY1 and PSY2, cluster analysis was performed based on their genome-wide sequences in comparison with the known S. lycopersicum cv. Red Setter and C. annuum cv. Zunla 1 PSY1 and PSY2 (Fig. 2). On the dendrogram, the pepper cultivars were expectedly grouped into two large clusters combining the sequences PSY1 and PSY2, respectively (see Fig. 2). Within each cluster, C. annuum accessions formed a single closely related subcluster with insignificant internal bootstrap values (17–50) and the only reliable combination of cv. Maria and Zheltyj buket based on PSY1. The S. lycopersicum species occupied the base branch in each of the clusters.

Thus, the identified PSY1 and PSY2 of nine pepper cultivars, which differ in a ripe fruit color, were highly similar in structure, which suggests that they may preserve the conserved key functions of phytoene synthases in the carotenoid biosynthesis.

**The content of chlorophylls and carotenoids in fruit pericarp during ripening**

The total content of chlorophylls and carotenoids was measured in fruit pericarp during development in the analyzed pepper cultivars (see Table 1). It was shown that unripe fruit of all cultivars (stage MF) contains comparable amounts of chlorophylls and carotenoids, which characterizes the fruit tissues as photosynthetic. In IR fruits, chlorophyll content decreased by 1.46–5.60 times, depending on the cultivar. In ripe fruits (RF stage), chlorophyll was found in significant quantities in cv. Sladkij shokolad, Shokoladnyj and Nesozrevayuschij, and in small quantities in Gogoshary and cv. Mechta hozyayki. There were no chlorophylls in ripe fruits of cv. Zheltyj buket, Karmin and Maria.

In immature fruits, the carotenoid content was the highest in the cv. Shokoladnyj and Sladkij shokolad (48.5 and 61.0 μg/g), while in the other cultivars it ranged from 6.8 (Gogoshary) to 27.3 μg/g (Zheltyj buket) (see Table 1). In ripe fruits, the primacy remained with the cultivars forming chocolate-colored fruits: the highest carotenoid content was detected in the cv. Shokoladnyj (1009.90 μg/g), while in the cv. Sladkij shokolad, it was reduced by 1.7 times, and in the red-fruited cv. Karmin and Maria – by 1.94 and 2.48 times, respectively (see Table 1). Ripe fruits of the remaining four cultivars accumulated significantly less carotenoids. However, a similar low carotenoid content did not provide the similar ripe fruit color: Zheltyj buket – yellow, Mechta hozyayki – red, Nesozrevayuschij and Gogoshary – green and red-green, respectively. At the same time, cv. Nesozrevayuschij and Gogoshary fruits had on average two times less carotenoids in comparison with the cv. Zheltyj buket and Mechta hozyayki fruits (see Table 1).

In accordance with the obtained biochemical data, it can be assumed that red-fruited cultivars synthesize red pigments typical for peppers – carotenoids capsanthin and capsorubin. In brown-fruited cultivars, the color may be formed by two components – red carotenoids and green chlorophylls. The yellow or green color of fruits at the stage of biological ripeness is most likely determined by the presence of yellow-colored carotenoids (lutein, zeaxanthin) and chlorophylls, respectively.

**PSY1 and PSY2 co-expression pattern in the fruit pericarp during ripening**

**Fig. 3.**
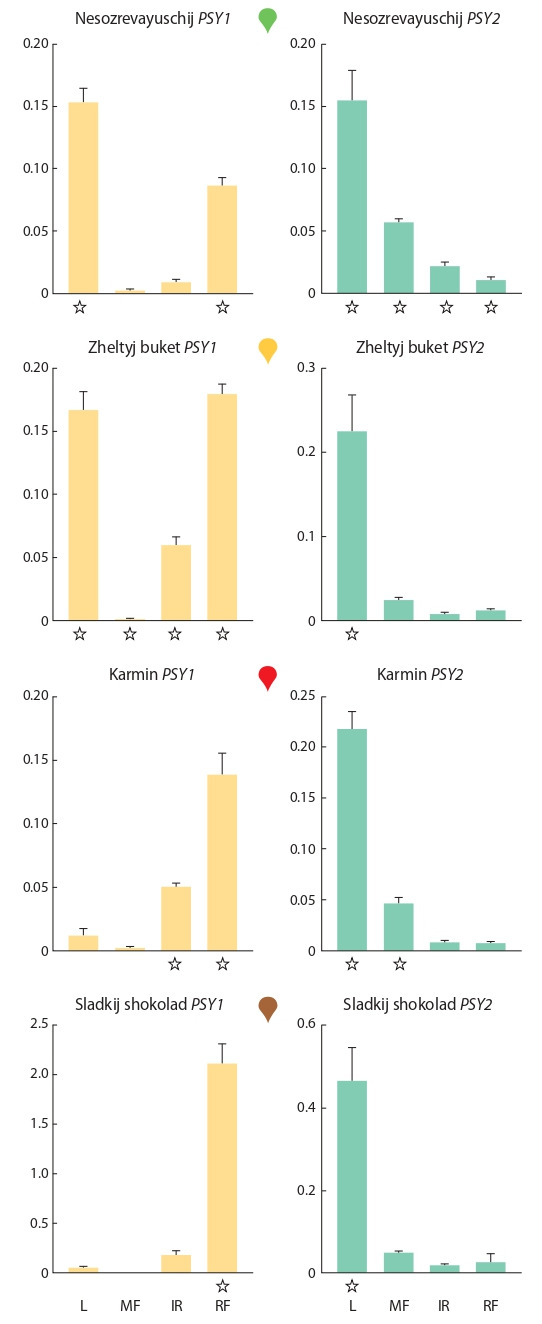
The PSY1 and PSY2 expression pattern in leaves (L) and fruits at three stages of ripening (MF – mature fruit, IR – intermediate ripe fruit, RF – ripe fruit) of C. annuum cultivars. Asterisks indicate gene expression values that differ significantly from its expression in all other tissues of the same accession.

Carotenoid accumulation in fruits is directly related to the PSY1 expression level (Meléndez-Martínez et al., 2010); however, although PSY2 is mainly expressed in photosynthetic tissues, its transcripts have also been found in fruits (Jang et al., 2020). In this study, PSY1 and PSY2 expression pattern was characterized in leaves and fruit pericarp (peel and pulp) at three ripening stages (MF, IR, RF) in four pepper cultivars (Fig. 3). The analysis included cv. Zheltyj buket, Sladkij shokolad, Karmin and Nesozrevayuschij, contrasting in the ripe fruit color (RF) – yellow, brown, dark red and green, respectively (see Table 1). Ripe fruits of cv. Karmin and Sladkij shokolad were characterized by a high carotenoid content (520.2 and 597.0 μg/g), while ripe fruits of cv. Nesozrevayuschij, and Zheltyj buket accumulated only 55.9 and 107.7 μg/g, respectively.

Phytoene synthase PSY2 is considered to be more specific for photosynthetic tissues (Giorio et al., 2008). In green unripe fruit (stage MF), all analyzed cultivars were characterized by the presence of chlorophylls – the highest in cv. Sladkij shokolad and the lowest in cv. Nesozrevayuschij. Surprisingly, only in these two cultivars, ripe fruits also contained chlorophyll (in the former it was 3 times higher than in the latter).

In the leaves, the PSY2 expression level in cv. Sladkij shokolad was 2–3 times higher than in the other three cultivars, in which the expression was comparable (see Fig. 3). Besides, PSY2 transcripts were detected in the fruit pericarp at all ripening stages in all analyzed cultivars. In the immature fruit pericarp (stage MF), PSY2 expression was approximately at the same level in cv. Nesozrevayuschij (0.057), Sladkij shokolad (0.050), and Karmin (0.046), while in cv. Zheltyj buket, it was twice lower (0.025) (see Fig. 3). In all analyzed cultivars, the PSY2 expression level decreased as fruits ripen. In ripe fruits (stage RF), the PSY2 level was similar in cv. Nesozrevayushchij, Zheltyj buket, and Karmin, and 2.3–3.5 times higher in cv. Sladkij shokolad (see Fig. 3).

It can be assumed that the presence of PSY2 expression in pepper ripe fruits is associated with the chloroplast preservation. This was evidenced by the chlorophyll presence in the ripe fruit pericarp, for example, in cv. Nesozrevayushchij and Sladkij shokolad (see Table 1). However, cv. Zheltyj buket and Karmin also showed PSY2 expression in ripe fruits, while no chlorophyll was found there. Thus, it can be assumed that PSY2 can function not only in chloroplasts, but also in chromoplasts. Earlier, using pepper cv. MicroPep Yellow as an example (with lack of PSY1 gene transcription), it was shown that the synthesis and accumulation of yellow pigments in fruit chromoplasts is associated with PSY2 expression (Jang et al., 2020).

The regulation of carotenoid biosynthesis and accumulation in pepper fruits is a complex process (Deruère et al., 1994; Kilcrease et al., 2015). In ripe fruits, carotenoids accumulate in chromoplasts in specialized globules, and if their formation is impaired, then carotenoids can be synthesized, but not accumulated (Osorio, 2019). Globule formation is controlled by the Orange protein, which at the same time prevents carotenoid degradation and stabilizes the phytoene synthase PSY activity (Osorio, 2019). Peel of cv. Nesozrevayuschij fruits, as they ripen, retained the green color, while the pulp color changes from light green to yellow-green. In accordance with this and in contrast to the other three analyzed cultivars, there was no significant increase in the carotenoid content (see Table 1), and the PSY1 transcription level in the ripe fruit of cv. Nesozrevayuschij was 1.6 and 2.1 times lower than that of cv. Karmin and cv. Zheltyj buket, respectively. It can be assumed that in cv. Nesozrevayuschij, a number of processes characteristic of fruit ripening, such as degradation of chlorophyll, transformation of chloroplasts into chromoplasts, and/or de novo chromoplast synthesis, are disturbed (Kilcrease et al., 2015; Berry et al., 2019). However, the fruits of cv. Nesozrevayuschij ripen (the seeds are fully formed and viable), although there is no noticeable change in the pericarp color. This confirms the previously shown lack of a relationship between fruit carotenogenesis and ripening (Fraser et al., 2007).

## Conclusion

Thus, in the present study, in nine C. annuum cultivars, differing in ripe fruit color, PSY1 and PSY2 genes encoding phytoene synthases were identified and characterized; the co-expression pattern of these genes in the vegetative and reproductive organs, as well as possible relationships of the expression level with the total carotenoid content were determined. A direct correlation was found between the PSY1 gene expression level and carotenoid pigmentation of the fruit during ripening. It was shown that in the cv. Nesozrevayuschij, the pericarp pigmentation pattern, typical for pepper fruits during ripening, is disturbed, which may be associated with blocks in the chromoplast formation.

## Conflict of interest

The authors declare no conflict of interest.
